# Effects of two sectional matrix systems on proximal contact tightness and mesiodistal dimension in class II composite restorations: an *in vitro* study

**DOI:** 10.3389/fdmed.2026.1932605

**Published:** 2026-08-12

**Authors:** Aqeel A. Noorudeen, S. Vidhyadhara Shetty, Nikhil Harikrishnan, Nihala Mariyam

**Affiliations:** Department of Conservative Dentistry and Endodontics, Yenepoya (Deemed to be University) Dental College, Mangaluru, India

**Keywords:** class II composite restoration, FDI criteria, mesiodistal dimension, proximal contact tightness, sectional matrix system, typodont model

## Abstract

**Objectives:**

To compare the effects of two sectional matrix systems on proximal contact tightness and mesiodistal (M-D) dimension in standardised Class II mesio-occlusal (MO) composite restorations.

**Methods:**

Twenty typodont mandibular first molar teeth (Nissin Dental Products Inc., Kyoto, Japan) received standardised Class II MO cavity preparations and were randomly allocated into two groups (*n* = 10 each). Group 1 received restorations with the myQuickmat Classico sectional matrix system (Polydentia SA, Mezzovico-Vira, Switzerland); Group 2 received restorations with the Septodont TDV Unimatrix R sectional matrix system (Septodont, Saint-Maur-des-Fossés, France). Anatomical plastic wedges (The Wedge, Polydentia SA, Mezzovico-Vira, Switzerland) were used gingivally in both groups. All restorations used Tetric N-Ceram nano-hybrid composite resin (Ivoclar Vivadent, Schaan, Liechtenstein). Proximal contact tightness was evaluated qualitatively using the FDI World Dental Federation clinical evaluation criteria for approximal anatomical form, assessed with dental floss and standardised stainless steel metal strips of graded thickness (25 and 50 μm). M-D dimension was measured at the occlusal and middle thirds using a digital Vernier calliper. The Shapiro–Wilk test confirmed normality of M-D data; group comparisons used the independent-samples t-test (M-D dimensions) or Mann–Whitney *U*-test (FDI scores). Significance was set at *p* < 0.05.

**Results:**

Group 1 demonstrated significantly greater M-D dimensions at the occlusal third (7.07 ± 0.054 mm vs. 6.70 ± 0.082 mm; *p* < 0.001) and the middle third (6.93 ± 0.060 mm vs. 6.67 ± 0.080 mm; *p* < 0.001). FDI scores for proximal contact tightness were also significantly superior in Group 1 (median 1, range 1–2) compared with Group 2 (median 2, range 2–3) (Mann–Whitney *U* = 9.00; *p* < 0.001).

**Conclusions:**

Within the limitations of this *in vitro* study, the myQuickmat Classico system (Polydentia SA, Mezzovico-Vira, Switzerland) produced significantly tighter proximal contacts and greater M-D dimensions compared with the Septodont TDV Unimatrix R system (Septodont, Saint-Maur-des-Fossés, France) in Class II MO composite restorations. These findings suggest that even within the sectional category, the choice of matrix system can meaningfully influence proximal restorative outcomes.

**Clinical relevance:**

Selection of an appropriate sectional matrix system may influence proximal contact quality in Class II composite restorations, with implications for food impaction prevention and long-term periodontal health. The present findings may assist clinicians in making evidence-informed choices between commercially available sectional systems.

## Introduction

The use of Class II composite resin restorations has been adopted as the gold standard for restoring proximal carious lesions in the posterior teeth, owing to advancements in the properties of materials, bonding techniques, and patient acceptance of aesthetic tooth-colored fillings. Even so, establishing a physiological proximal contact is still one of the most technique-sensitive procedures in direct dental restoration (1). Poorly established proximal contacts predispose patients to food impaction, dental biofilm formation, secondary caries, and periodontal deterioration. Such complications compromise the long-term prognosis of the restored tooth and adjacent periodontal tissues (2, 3).

Matrix systems play a very important role in the determination of the quality of proximal contact. This is because matrix systems act as a temporary barrier wall which helps to confine the restorative material, establish the emergence contour, and when used with an effective tooth separation system, can compensate for the bulkiness of the band. Circumferential matrix systems, designed for amalgam restorations, have shown to be less effective in composite resin restorations and have produced flat proximal contacts, poor separation, and weak contacts (4, 5). Several systematic reviews have examined this comparison, though from somewhat different angles. Alshardan et al. (6) and Kamble et al. (7), both synthesising clinical-trial evidence, concluded that sectional matrix systems produce more favourable proximal contacts than circumferential systems; Drouri et al. (8) reached a similar conclusion while additionally examining how matrix design interacts with the physical behaviour of the composite material itself.

The sectional matrix system uses pre-formed stainless steel bands, wedges, and separators, which usually include nickel-titanium separators, to provide a precisely directed lateral pressure on the interproximal contact point. This pressure provides a counter-pressure for the band thickness and allows the tooth next to the restoration to move back into place when the separator is removed, thus achieving better contact than circumferential matrices can achieve (9). Previous systematic reviews have confirmed this benefit, and clinical studies have demonstrated the superiority of contact tightness by sectional ring systems (5, 10).

Although the advantages of the sectional system over the circumferential system have been proven before, there have not been many studies comparing various sectional matrices. Differences in the geometry of the pre-shaped band, angulations of the ring tines, the magnitude of the separating forces, and the width of the bands might result in differences in the quality of proximal contacts and the mesiodistal (M-D) dimension accuracy of the finished restoration—a characteristic highly correlated with arch integrity (11–13). To date, there have been no studies that have compared the two sectional matrices, myQuickmat Classico (Polydentia SA, Mezzovico-Vira, Switzerland) and Septodont TDV Unimatrix R (Septodont, Saint-Maur-des-Fossés, France). According to manufacturer specifications, these two systems differ primarily in separation-ring design: the myQuickmat Classico uses a nickel-titanium separation ring (myRing Classico), whereas the Septodont TDV Unimatrix R offers interchangeable Hard and Soft reinforced rings fitted with triangular silicone tips, with the Hard reinforced ring used in this study. Both systems use identically specified 0.03 mm matrix bands. This difference in ring material and force delivery provided the rationale for directly comparing these two specific systems.

Proximal contact tightness has been quantified in the literature using force-based (universal testing machine, Tooth Pressure Meter) and qualitative (FDI criteria, dental floss resistance) approaches. The FDI World Dental Federation criteria provide a validated, clinically intuitive ordinal framework that grades proximal contact from physiologically optimal through unacceptable (14). Simultaneously assessing M-D dimension by digital calliper allows quantification of the final restoration volume at the proximal contact level — an indirect index of band adaptation efficacy (15).

The present study aimed to evaluate and compare the effects of the myQuickmat Classico and the Septodont TDV Unimatrix R sectional matrix systems (Septodont, Saint-Maur-des-Fossés, France) on proximal contact tightness (FDI score) and M-D dimension in standardised Class II MO typodont composite restorations. The null hypothesis was that neither system would produce a statistically significant difference in either outcome parameter.

## Methods

### Study design and sample size

This parallel-group *in vitro* study was conducted at the Department of Conservative Dentistry and Endodontics, Yenepoya Dental College, Mangaluru. Sample size was calculated using G*Power (version 3.1.9.7) for an independent-samples *t*-test with *α* = 0.05, powe*r* = 95%, and an effect size of 1.72 derived from mean proximal contact tightness values (sectional matrix: 4.22 ± 0.90; circumferential matrix: 3.03 ± 0.39) reported by Tolba et al. (4) This yielded a total sample of 20, allocated equally between two groups (*n* = 10 per group).

### Specimen preparation

Twenty typodont mandibular first permanent molar teeth (Nissin Dental Products Inc., Kyoto, Japan) were selected to ensure morphological standardisation and eliminate inter-specimen biological variability, consistent with previous matrix system investigations (4, 12). All teeth were retained in their original phantom head sockets throughout experimentation to standardise operative positioning. As the specimens were identical replicas produced to the manufacturer's design tolerances rather than extracted natural teeth, pre-preparation mesiodistal dimensions were assumed to be equivalent across specimens and were not independently measured before cavity preparation. Standardised Class II mesio-occlusal (MO) cavity preparations were performed by a single calibrated operator using a high-speed handpiece and diamond burs under continuous water irrigation. Each cavity comprised two distinct components with the following standardised dimensions. The occlusal component had a pulpal depth of 1.5 mm. The proximal box had a buccolingual width of 4 mm, an occlusogingival height of 3 mm, and a mesiodistal depth of 1.5 mm at the proximal wall.

### Randomisation and group allocation

Following preparation, specimens were randomly allocated using a simple random sequence to one of two groups:

**Group 1 (*n*** **=** **10):** myQuickmat Classico sectional matrix system (Polydentia SA, Mezzovico-Vira, Switzerland).

**Group 2 (*n*** **=** **10):** Septodont TDV Unimatrix R sectional matrix system (Septodont, Saint-Maur-des-Fossés, France).

### Restorative protocol

**Figure 1:**
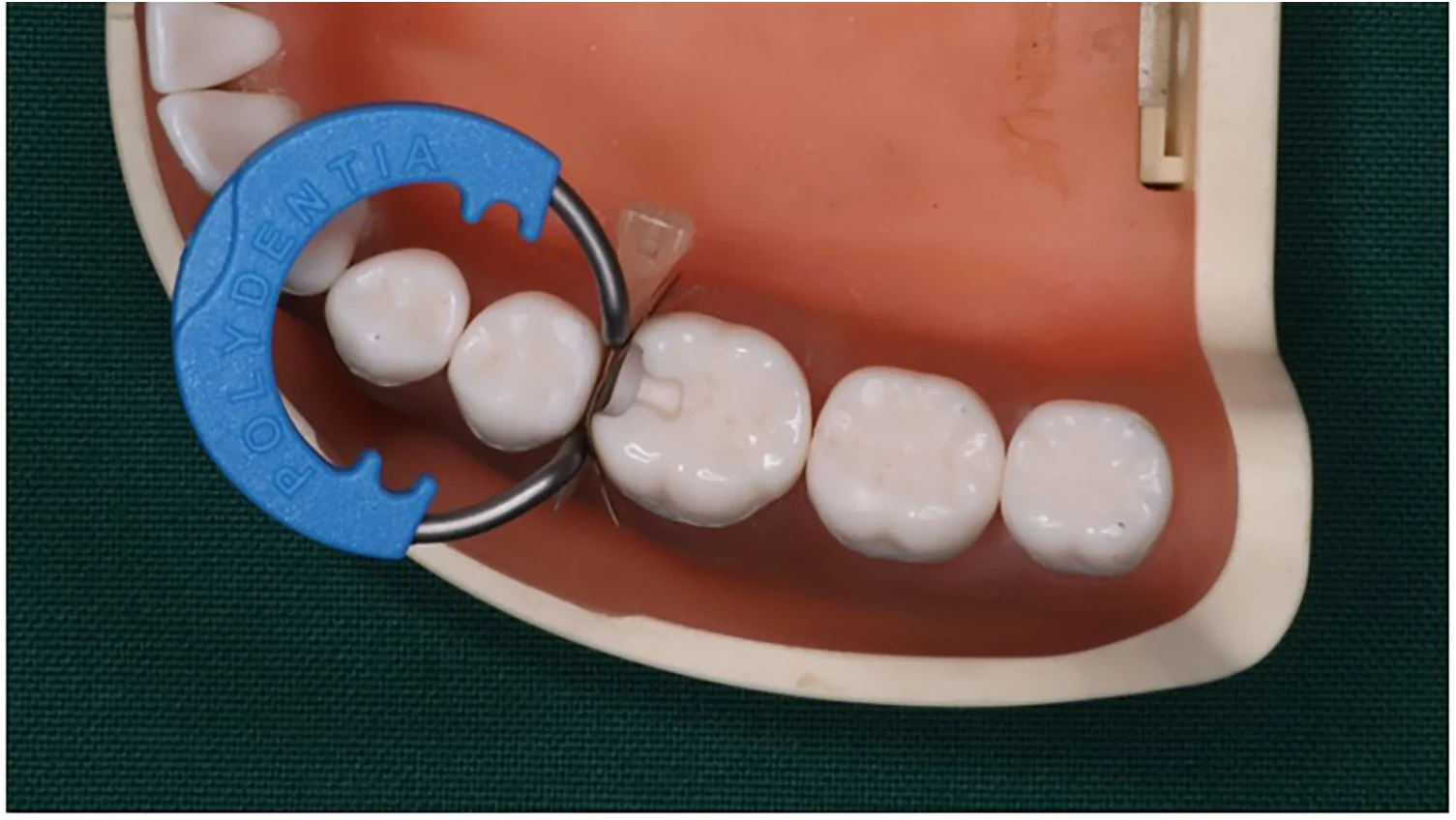
Application of sectional matrix system (myQuickmat classico, (polydentia SA, mezzovico-Vira, Switzerland)) at the mesial proximal box (group 1).

**Figure 2:**
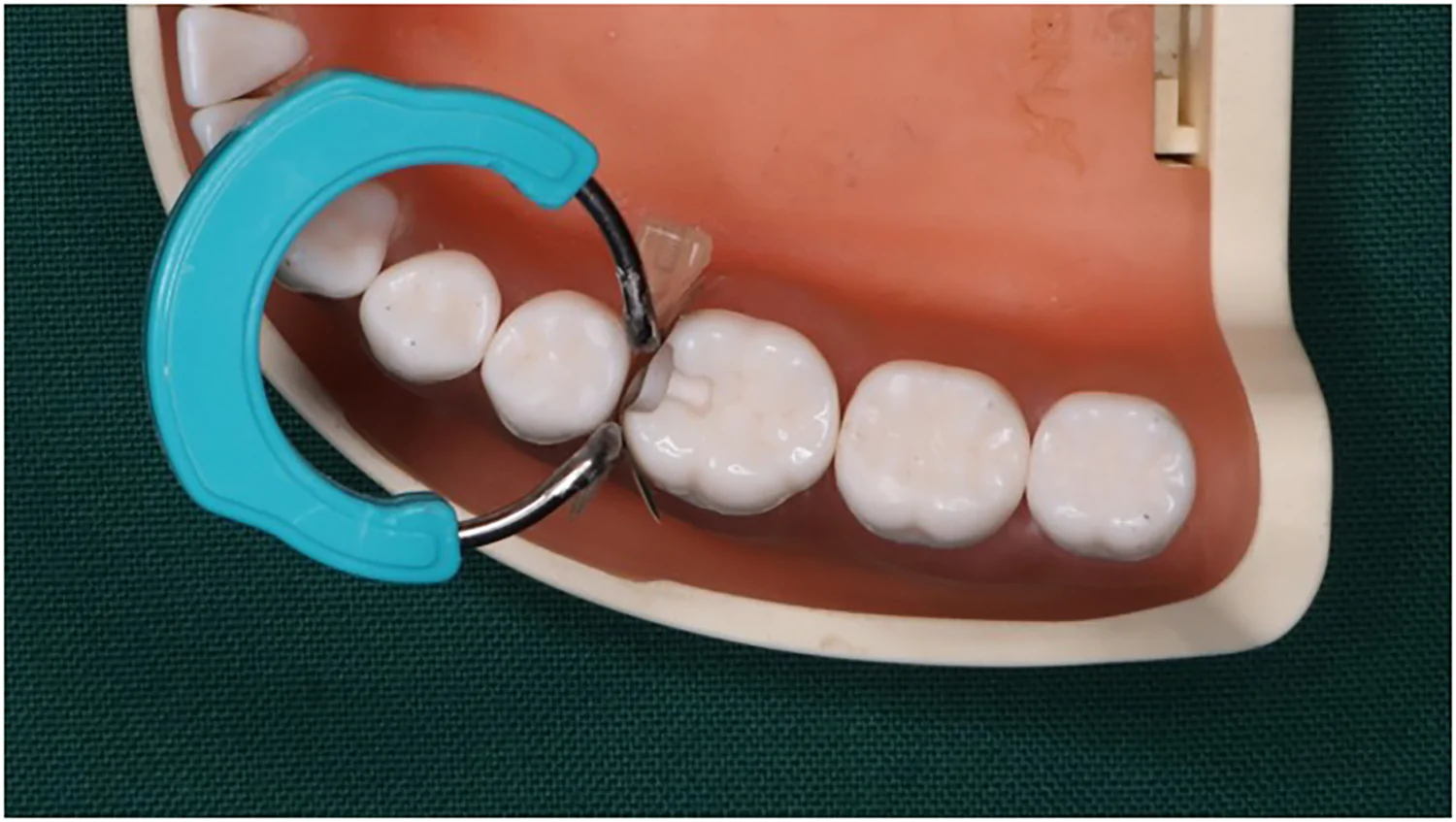
Application of sectional matrix system (septodont TDV unimatrix R system (septodont saint-maur-des-fossés, France)) at the mesial proximal box (group 2).

The pre-contoured stainless steel sectional bands provided with each of the two systems was utilised without any modification and burnishing, in order to have standardised band dimensions. The sectional band was positioned at the mesial proximal box, while the separation ring was placed interproximally. Anatomical plastic wedges (The Wedge, Polydentia SA, Mezzovico-Vira, Switzerland) were placed gingivally in both groups in order to adapt the matrix band at the gingival margin of the cavity (Figures 1, 2).

Tetric N-Bond Universal adhesive (Ivoclar Vivadent AG, Schaan, Liechtenstein) was applied per manufacturer's instructions and light-cured for 10 s using an LED unit (Woodpecker ILED Plus, Guilin Woodpecker Medical Instrument Co., Ltd., Guilin, China) at ≥1,500 mW/cm^2^. Tetric N-Ceram nano-hybrid composite resin (Ivoclar Vivadent AG, Schaan, Liechtenstein) was placed incrementally in three equal increments, each light-cured for 20 s. All procedures were performed by a single operator to minimise inter-operator variability.

### Outcome assessments

#### Mesiodistal dimension

The maximum M-D dimension of each restored tooth was measured using a calibrated digital Vernier calliper (resolution 0.01 mm) at two standardised levels: (i) the occlusal third, and (ii) the middle third of the proximal surface. Three sequential measurements were recorded at each level, and the mean value was used in analysis. A single operator performed all measurements to control for inter-rater variability.

#### Proximal contact tightness (FDI score)

Proximal contact tightness was evaluated qualitatively using the FDI World Dental Federation clinical evaluation criteria for the approximal anatomical form of direct restorations (14). Dental floss and a standardised stainless steel metal strip were each passed through the proximal contact space under standardised conditions, and the resistance encountered during insertion was graded. This approach is consistent with previous *in vitro* and *in vivo* studies employing FDI criteria for proximal contact assessment (14, 16). (Table 1).

**Table 1:** FDI world dental federation criteria for the evaluation of proximal contact tightness (category F3), adapted from hickel et al. (14).

Score	Clinical grade	Criteria (clinical description)	Management
1	Clinically excellent/very good *Sufficient*	Ideal proximal contact point: the 25 μm metal blade can pass through the proximal contact with resistance. No inflammation of the gingiva or periodontium attributable to the proximal restoration. No food impaction.	Review/Monitor
2	Clinically good *Sufficient*	Slightly weak proximal contact point: the 50 μm metal blade can pass through the proximal contact. No inflammation of the gingiva or periodontium attributable to the proximal restoration. No food impaction.	Review/Monitor
3	Clinically satisfactory *Sufficient*	Either of the following: (a) Oversized contact point/excess restorative material: the 25 μm metal blade cannot pass through the proximal contact and inflammation of the gingiva or periodontium attributable to the proximal restoration is present; refurbishment is possible. (b) Severely weak contact point: the 100 μm metal blade can pass through the proximal contact but there is no inflammation of the gingiva or discomfort.	Refurbishment
4	Clinically unsatisfactory *Partially insufficient*	Severely weak proximal contact point: the 100 μm metal blade can pass through the proximal contact, or an unintended interlocked contact point is present. Inflammation of the gingiva or periodontium attributable to the proximal restoration and/or food impaction is evident. Repair is possible.	Repair
5	Clinically poor *Entirely insufficient*	Severely weak proximal contact point: the 100 μm metal blade can easily pass through the proximal contact, or an unintended interlocked contact point is present (impossible for the blade to pass). Inflammation of the gingiva or periodontium attributable to the proximal restoration and/or food impaction is evident. Repair is not possible or reasonable.	Replacement

FDI, Fédération Dentaire Internationale; μm, micrometres.

### Statistical analysis

Data were entered in Microsoft Excel and analysed using Jamovi statistical software (version 2.6.44; The jamovi project, Sydney, Australia). Continuous variables were summarised as mean, median, standard deviation (SD), minimum, maximum, 25th percentile (Q1), and 75th percentile (Q3). The Shapiro–Wilk test assessed distributional normality. M-D dimension variables (occlusal third: *W* = 0.956, *p* = 0.744 [Group 1]; *W* = 0.926, *p* = 0.408 [Group 2]; middle third: *W* = 0.944, *p* = 0.600 [Group 1]; *W* = 0.962, *p* = 0.813 [Group 2]) satisfied the normality assumption (*p* > 0.05) and were compared between groups using the independent-samples Student's *t*-test. FDI scores violated normality (*W* = 0.594 and 0.640, *p* < 0.001 for Groups 1 and 2, respectively) and were compared using the Mann–Whitney *U*-test. A *p*-value < 0.05 was considered statistically significant.

## Results

Detailed results are presented in Tables 2, 3.

**Table 2:** Descriptive statistics and shapiro–wilk normality test results.

Variable	Group	Mean	Median	SD	Min	Max	W	*p*	Q1	Q3
FDI Score*	myQuickmat Classico	1.30	1.00	0.483	1	2	0.594	<0.001	1.00	1.75
	Septodont TDV Unimatrix R	2.40	2.00	0.516	2	3	0.640	<0.001	2.00	3.00
M-D Dimension, Occlusal Third (mm)	myQuickmat Classico	7.07	7.08	0.054	6.99	7.15	0.956	0.744	7.03	7.12
	Septodont TDV Unimatrix R	6.70	6.72	0.082	6.58	6.81	0.926	0.408	6.63	6.76
M-D Dimension, Middle Third (mm)	myQuickmat Classico	6.93	6.94	0.060	6.84	7.01	0.944	0.600	6.88	6.98
	Septodont TDV Unimatrix R	6.67	6.67	0.080	6.54	6.79	0.962	0.813	6.60	6.73

Group 1: myQuickmat Classico (Polydentia SA); Group 2: Septodont TDV Unimatrix R. SD = standard deviation; Q1 = 25th percentile; Q3 = 75th percentile; *W* = Shapiro–Wilk statistic; *p* = Shapiro–Wilk *p*-value.

*FDI Score was non-normally distributed in both groups; ordinal data, Mann–Whitney *U*-test applied for group comparison. M-D = mesiodistal.

**Table 3:** Inferential statistics comparing group 1 (myQuickmat classico) and group 2 (septodont TDV unimatrix R).

Variable	Test	Statistic	df	*p*-value
FDI Score (ordinal; non-normal)	Mann–Whitney U	9.00	—	<0.001*
M-D Dimension, Occlusal Third (mm)	Student's *t*-test	12.01	18	<0.001*
M-D Dimension, Middle Third (mm)	Student's *t*-test	8.27	18	<0.001*

df = degrees of freedom; * = statistically significant (*p* < 0.05); — = not applicable.

Mann–Whitney *U*-test applied for FDI Score; Student's t-test applied for normally distributed M-D dimension variables.

### Mesiodistal dimension

Group 1 (myQuickmat Classico (Polydentia SA, Mezzovico-Vira, Switzerland)) produced greater mean M-D dimensions than Group 2 (Septodont TDV Unimatrix R (Septodont, Saint-Maur-des-Fossés, France)) at both the occlusal third (7.07 ± 0.054 mm vs. 6.70 ± 0.082 mm) and the middle third (6.93 ± 0.060 mm vs. 6.67 ± 0.080 mm), and these differences were statistically significant (Table 2). Within both groups, M-D dimensions were marginally greater at the occlusal third compared with the middle third.

### Proximal contact tightness

Group 1 (myQuickmat Classico) produced superior proximal contact tightness compared with Group 2 (Septodont TDV Unimatrix R (Septodont, Saint-Maur-des-Fossés, France)), and this difference was statistically significant (Table 2). The majority of Group 1 restorations were graded as FDI Score 1 (optimal contact), with a median score of 1 and a range of 1 to 2. In contrast, Group 2 restorations received predominantly FDI Scores of 2 and 3, with a median of 2 and a range of 2 to 3. No restoration in Group 2 achieved the optimal FDI Score of 1.

## Discussion

This *in vitro* study compared the myQuickmat Classico (Polydentia SA, Mezzovico-Vira, Switzerland) and Septodont TDV Unimatrix R sectional matrix systems (Septodont, Saint-Maur-des-Fossés, France) in standardised Class II MO composite restorations using two outcome parameters: proximal contact tightness (FDI score) and M-D dimension. The myQuickmat Classico system (Polydentia SA, Mezzovico-Vira, Switzerland) produced statistically significant advantages in both parameters under the tested laboratory conditions, leading to rejection of the null hypothesis; whether these differences translate into clinically meaningful benefits remains to be confirmed *in vivo*. To the authors' knowledge, this is the first study to directly compare these two commercially available sectional systems on both contact tightness and dimensional accuracy simultaneously.

The FDI scoring results revealed a clear disparity in contact quality between the groups. Group 1 (myQuickmat Classico (Polydentia SA, Mezzovico-Vira, Switzerland)) consistently produced physiologically optimal contacts (median FDI score 1), while Group 2 (Septodont TDV Unimatrix R (Septodont, Saint-Maur-des-Fossés, France)) yielded predominantly acceptable-to-deficient outcomes (median score 2, with scores reaching 3 in some specimens). These findings align with those of Sankhyan et al. (16), who employed FDI criteria in an *in vivo* study comparing three matrix systems and reported that sectional systems with effective separation mechanisms — Garrison (Garrison Dental Solutions, Spring Lake, Michigan, USA) and Bioclear (Bioclear Matrix Systems, Tacoma, Washington, USA) — produced significantly superior proximal contact and contour scores compared with the circumferential Tofflemire matrix. The graduation of FDI scores in the current study suggests that system-specific mechanical differences are sufficient to shift restorations from optimal to clinically acceptable or unacceptable contact categories — distinctions with direct periodontal significance.

The M-D dimension findings corroborate and extend the FDI results. Group 1 produced larger M-D dimensions at both the occlusal third (7.07 vs. 6.70 mm) and middle third (6.93 vs. 6.67 mm), with the between-group differences of 0.37 mm and 0.26 mm, respectively, each achieving high statistical significance (*p* < 0.001). A greater M-D dimension at the proximal contact level reflects more effective matrix adaptation against the adjacent tooth surface, achieved through stronger and more sustained interproximal separation. This dimensional recreation is not merely a geometric curiosity: a reduced or incompletely restored proximal bulk leaves a residual gap or under-contoured contact area that has been directly linked to food impaction, interproximal plaque retention, and consequent secondary caries and periodontal breakdown (2, 3), so that a larger, more completely restored M-D dimension can reasonably be interpreted as a proxy for a more clinically favourable restoration outcome rather than an isolated dimensional finding. Kumari et al. (15) demonstrated analogous dimensional differences when comparing the Palodent and Palodent Plus systems, reporting that superior mesiodistal diameters correlated with better contact quality. Their study also highlighted that flatter band profiles — analogous to the lesser mesiodistal bulk observed in Group 2 of the present study — were associated with less favourable proximal form. The present findings are therefore mechanistically consistent with that body of evidence.

One plausible biomechanical explanation for the difference observed between the two systems relates to the design and force-delivery characteristics of their respective separation rings. According to manufacturer specifications, the myQuickmat Classico uses a nickel-titanium ring, whereas the Septodont TDV Unimatrix R offers interchangeable Hard/Soft reinforced rings with silicone tips, with the Hard reinforced ring used in this study; both systems use identically specified (0.03 mm) matrix bands, so band thickness itself is not proposed as a differentiating factor. It is conceivable that the difference in ring material and force delivery could influence the magnitude of interproximal separation and, in turn, the rebound effect that tightens the contact after ring removal, a mechanism broadly consistent with observations reported by Wirsching et al. (5) and Saber et al. (11) in other matrix systems. This explanation remains speculative, however: no independent force measurements of the rings, nor independent dimensional verification of the bands used on the study specimens, were performed in the present study, and the manufacturer-reported specifications above may not fully reflect the mechanical behaviour of the components under clinical loading. Direct force and dimensional measurements would be required before this mechanism could be considered more than a hypothesis.

The broader literature consistently supports the superiority of sectional ring-assisted systems in achieving proximal contact tightness. Anantula et al. (10) synthesised available evidence across multiple matrix types and concluded that the combination of sectional matrices and separation rings produced proximal contact forces closest to those of intact unrestored teeth. The present data extend this to intra-category comparisons, demonstrating that even among sectional systems, design-level differences produce measurable and statistically significant outcome variation. This is clinically important because dentists making system-level selections within the sectional category cannot assume equivalent performance.

The qualitative nature of FDI scoring is a recognised limitation of this outcome measure. While validated and widely adopted in clinical research (14), ordinal scoring is inherently less sensitive than quantitative force-based measurements obtained with a universal testing machine or Tooth Pressure Meter. It is possible that differences in contact force between the groups were greater than the FDI scale resolution captures. Future investigations pairing FDI scoring with objective force measurement would provide complementary quantitative confirmation of the present findings.

Further limitations include the *in vitro* typodont model, which does not replicate the compliance of the periodontal ligament, saliva contamination, thermal cycling, and functional occlusion loading, which affect contact stability clinically. Because the typodont model lacks periodontal ligament (PDL) resilience, it cannot reproduce the small, elastic tooth displacement that PDL compression permits clinically; this rebound is thought to contribute to how sectional separation rings achieve final contact tightness, and its absence here means the present model may not fully represent the magnitude of this effect *in vivo*. In addition, because pre-preparation mesiodistal dimensions of the unprepared typodont teeth were not independently measured, group equivalence before restoration relied on the manufacturer's standardisation of the typodont replicas rather than on direct baseline verification; this should be regarded as an assumption rather than a confirmed finding. The number of participants (*n* = 10 per group), which is adequate for the primary test, lacks generalisability. Only one cavity type (Class II MO in mandibular first molars), one composite material, and one adhesive system were evaluated; generalisability to other cavity configurations, tooth types, and material combinations requires further investigation. Blinding of the operator was not feasible due to the nature of procedures involved in this study; nevertheless, the use of objective calliper readings and standardised FDI classification criteria were used to reduce subjectivity. Thermocycling and mechanical loading were not done; therefore, the findings represent only immediate post-restoration laboratory outcomes.

Clinical relevance of the variation among sectional systems within each category reinforces the requirement of evidence-based system selection. With increasing availability of newer sectional systems as well as modified versions of existing sectional systems in terms of varying band geometry, wedge systems, and ring systems, the *in vitro* comparison data like that presented here can serve as a useful basis for the evidence. Clinical trials have to be conducted to confirm if the dimensional and contact benefits demonstrated in laboratory conditions will result in long-term clinical outcomes, including interproximal plaque control, secondary caries incidence, and restoration longevity.

## Conclusions

Within the limitations of this *in vitro* study, the myQuickmat Classico sectional matrix system (Polydentia SA, Mezzovico-Vira, Switzerland) provided significantly greater proximal contact tightness and M-D dimensions of standardised Class II MO composite direct restorations than the Septodont TDV Unimatrix R sectional matrix system (Septodont, Saint-Maur-des-Fossés, France). Design-related differences among sectional matrix systems may be sufficient to produce measurable differences in laboratory outcome measures of proximal restorations, although confirmation in a clinical setting is required before these findings can be regarded as clinically significant. Clinicians should consider system-specific performance evidence when selecting sectional matrix systems for Class II composite restorations.

## Data Availability

The original contributions presented in the study are included in the article/Supplementary Material, further inquiries can be directed to the corresponding author/s.
